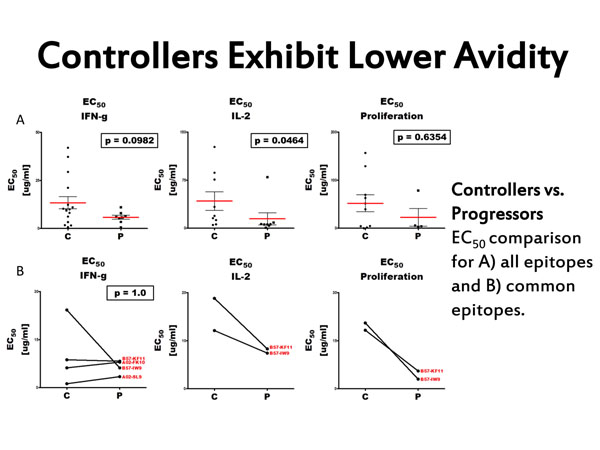# Role of functional avidity in HIV-specific memory CD8 T cell effector functions

**DOI:** 10.1186/1742-4690-9-S1-P26

**Published:** 2012-05-25

**Authors:** Tiffany Lemon, Donna Alvino, Zaza Ndhlovu, Bruce Walker

**Affiliations:** 1 Louisiana State University - Dept of Biological Sciences, Baton Rouge, USA

## 

CD8+ T cells provide protective antiviral defense in HIV-1 infection. Although studies demonstrate which effector functions are employed, knowledge of the underlying mechanisms is lacking and inconclusive. Here, we investigate the functional avidity of CD8+ T cells, based on cytokine secretion and proliferation, to compare the effective antigen concentration required to induce each response. Our preliminary data suggests that the functional avidity of CD8+ T cells differs based on the effector function used for measurement, indicating that the requirements for activation differ within a single CD8+ profile. We also found that elite controllers, individuals who control the virus without antiretroviral treatment, require lower avidity interactions than chronic progressors.

These and future results will help to determine optimal doses of antigens for the induction of effective responses in new vaccine formulations.

**Figure 1:**